# Efficiency and safety of HAIC combined with lenvatinib and tislelizumab for advanced hepatocellular carcinoma with high tumor burden: a multicenter propensity score matching analysis

**DOI:** 10.3389/fphar.2024.1499269

**Published:** 2025-01-07

**Authors:** Zhonghua Zhao, Xiongying Jiang, Shiping Wen, Yanzhang Hao

**Affiliations:** ^1^ Department of Oncology, Binzhou Medical University Hospital, Binzhou, Shandong, China; ^2^ Department of Interventional Radiology, Sun Yat-sen Memorial Hospital, Sun Yat-sen University, Guangzhou, China; ^3^ Department of Minimally Invasive Interventional Therapy, State Key Laboratory of Oncology in South China, Guangdong Provincial Clinical Research Center for Cancer, Sun Yat-Sen University Cancer Center, Guangzhou, China

**Keywords:** hepatocellular carcinoma, hepatic arterial infusion chemotherapy, tyrosine kinase inhibitors, programmed cell death protein-1 inhibitors, propensity score matching

## Abstract

**Purpose:**

The present work focused on assessing whether hepatic arterial infusion chemotherapy (HAIC) combined with lenvatinib and tislelizumab was safe and effective on advanced hepatocellular carcinoma (HCC) showing high tumor burden.

**Methods:**

In the present multicenter retrospective study, treatment-naive advanced HCC patients (BCLC stage C) showing high tumor burden (maximum diameter of intrahepatic lesion beyond 7 cm) treated with lenvatinib and tislelizumab with or without HAIC were reviewed for eligibility from June 2020 to June 2023. Baseline differences between groups were mitigated by propensity score matching (PSM). Our primary endpoint was overall survival (OS); and secondary endpoints included adverse events (AEs), progression-free survival (PFS), disease control rate (DCR) and objective response rate (ORR) according to RECIST 1.1 criteria, respectively.

**Results:**

After eligibility reviewed, total 162 patients treated with lenvatinib and tislelizumab were enrolled: 63 patients with HAIC (HTP group), and the remaining 99 patients without HAIC (TP group). After PSM 1:1, 47 cases were evenly divided into each group. Of them, HTP group showed significant prolonged median OS compared with TP group (16.6 versus 21.0 months; hazard ratio [HR]: 0.26, 95% confidence interval [CI]: 0.35–0.98; *p* = 0.039), and median PFS of HTP group was also prolonged (8.9 versus 11.6 months; HR: 0.55, 95% CI: 0.34–0.87; *p* = 0.010). Higher DCR and ORR could be observed in HTP relative to TP groups (ORR: 53.2% versus 17.0%, *p* < 0.001; DCR: 87.2% versus 61.7%, *p* = 0.004). The severe AEs (grade 3/4) and all grades were comparable between the groups, while all of these AEs could be controlled, and AEs of grade 5 were not reported.

**Conclusion:**

HAIC combined with lenvatinib and tislelizumab is the candidate treatment for advanced HCC patients because of its improved prognosis and acceptable safety.

## 1 Introduction

Hepatocellular carcinoma (HCC) ranks the 6^th^ place among cancers with regard to its morbidity and the 3^rd^ place among factors inducing cancer-associated mortality worldwide in 2022, and the harm is even greater in China (Bray et al., 2024). The main reason is that the diagnosis in over 70% cases is made at the advanced stage in China, when radical treatment is unfeasible, resulting in extremely poor prognosis (Younossi et al., 2023). In the past decades, lots of novel therapy methods have been developed for cancer treatment, including nanotechnology, targeted therapy, immunotherapy and so on (Zhu et al., 2024; Liu et al., 2023; Dai et al., 2024). As recommended by the Barcelona Clinic Liver Cancer (BCLC) classification system, systemic treatment with immunotherapy and targeted therapy can be applied in advanced HCC, and the life expectancy has obviously improved (Reig et al., 2022).

Sorafenib has been recommended to be a first-line treatment for advanced HCC from 2008, which is effective in the SHARP and Oriental clinical trial (Llovet et al., 2008; Cheng et al., 2009). Until 2017, lenvatinib shows comparable overall survival (OS) and significantly improved progression-free survival (PFS), objective response, time to progression (TTP), as well as postponed life quality decline to sorafenib among untreated, non-resectable HCC cases (Kudo et al., 2018). Based on the result, lenvatinib has become a novel preferred option for advanced HCC. Besides, multiple programmed cell death protein 1 (PD-1) inhibitors gain approval for HCC, including first-line or second-line therapy options. Among them, tislelizumab is promising in treating HCC, and exhibited durable antitumor effect and favorable OS among advanced HCC cases from the front-line cohort in RATIONALE 301 study, comparing to sorafenib (Qin et al., 2023).

Next, with the positive result of the IMbrave150 study, targeted therapy combined with immunotherapy is becoming a preferred option for advanced hepatocellular carcinoma (Finn et al., 2020a). Except for bevacizumab, tyrosine kinase inhibitors (TKIs) combined with immunotherapy have also undergone extensive clinical trials and practice. Lenvatinib combined with pembrolizumab as the first-line therapy displays good antitumor effect in the KEYNOTE-524 phase 1b study, the median OS was 22.0 months, and the median PFS was 8.6 months, accompanied by controllable safety (Finn et al., 2020b). Unfortunately, though LEAP-002 phase III study demonstrated that lenvatinib combined with pembrolizumab could significantly improve prognosis, it could not reach specific significance in terms of prolonged OS and PFS compared with lenvatinib plus placebo, indicating that the therapeutic effect of combination therapy needs to be further enhanced (Llovet et al., 2023).

FOLFOX-based hepatic artery infusion chemotherapy (HAIC) can effectively treat advanced HCC, which can substantially increase OS in comparison with sorafenib (Lyu et al., 2022). HAIC can transfer sustained high drug concentration to the tumors, and induce the great local anticancer efficacy, resulting in effectively shrinking intrahepatic lesions, especially for those with high tumor burden (Chen et al., 2023). In addition, it has reported that chemotherapy can exert synergistic anti-tumor effects with immunotherapy and targeted therapy in various types of cancer (Ye et al., 2023; Tubridy et al., 2024), and many studies have also demonstrated that HAIC combined with PD-1 inhibitors and TKIs can improve survival outcomes for advanced HCC (Zhang et al., 2023; Lin et al., 2023). However, it remains unclear whether the triple combination therapy is safe and effective on advanced HCC with high tumor burden.

Therefore, the present work focused on investigating whether HAIC combined with lenvatinib and tislelizumab versus lenvatinib plus tislelizumab was effective and safe in the treatment of advanced HCC with high tumor burden.

## 2 Methods

### 2.1 Participants

Advanced (BCLC stage C) HCC cases receiving lenvatinib and tislelizumab treatment from June 2020 to June 2023 were enrolled into the present retrospective study and divided into HTP group (triple combination of HAIC, lenvatinib and tislelizumab) and TP group (lenvatinib and tislelizumab) according to treatment option.

Patients below were included: (1) those with the age of 18–75 years; (2) those with radiological or pathological diagnosis of HCC in line with guidelines of the American Association for the Study of Liver Diseases (AASLD); (3) BCLC C stage, either with extrahepatic metastasis or portal vein thrombus (PVTT); (4) maximum diameter of intrahepatic lesion beyond 7cm; (5) no previous anti-HCC therapy; (6) Child-Pugh class A or B, ALBI class 1 or 2, and Eastern Cooperative Oncology Group Performance Status score (ECOG PS) 0 or 1; (7) without additional malignant diseases within 5 years; (8) received at least six cycles of tislelizumab, 3 months of lenvatinib, and two cycles of HAIC in the HTP group; (9) at least 12 months from enrollment to cut-off time; and (10) sufficient follow-up and medical data. Patients below were excluded: (1) pathologically diagnosed as fibrolamellar HCC, sarcomatous HCC, or combined hepatocellular cholangiocarcinoma (HCC-CC); (2) active upper gastrointestinal bleeding or coagulation dysfunction; (3) therapy regimen discontinued or changed with no appropriate reason; and (4) without informed consent. Imaging examinations like enhanced computed tomography (CT) and magnetic resonance imaging (MRI), and laboratory test results were obtained in 1 week prior to initiating treatment.

This work was approved by ethics committee of our institutions. This study did not require informed consent because of its retrospective design.

### 2.2 Treatment procedures

HAIC was performed by experienced interventional physicians at each center. The detailed procedure of HAIC with oxaliplatin, fluorouracil, and leucovorin (FOLFOX) combination therapy was described previously (Lyu et al., 2022; Lyu et al., 2018a; Lyu et al., 2018b). To be specific, the tumor-feeding branch of hepatic artery was inserted with a catheter tip selectively according to the tumor size, location, and arterial supply. The following regimen was administered: oxaliplatin (CENEXI-Laboratoires THISSEN S.A.) (130 mg/m^2^, 0–2 h on day 1), leucovorin (Qilu Pharmaceutical Co., Ltd.) (200 mg/m^2^, 2–4 h on day 1), fluorouracil (Qilu Pharmaceutical Co., Ltd.) (400 mg/m^2^ bolus within 15 min, and 2,400 mg/m^2^ > 46 h on days 1 and 2). Repetitive HAIC was determined to perform at intervals of 3 weeks with no more than 8 cycles according to operators’ evaluation.

Patients in both groups received oral administration of lenvatinib (Eisai Co., Ltd.) at 8 mg (≤60 kg) or 12 mg (>60 kg) in line with body weight, and 200 mg tislelizumab (BeiGene., Ltd.) intravenously every 3 weeks. Lenvatinib administration was conducted on day 1 during HAIC, whereas tislelizumab was given through intravenous injection on day 2 just after HAIC was completed in HTP group, while they were admitted at the same day in the TP group. If patients were intolerable due to the toxicities, lenvatinib or tislelizumab was reduced or discontinued until the disappearance of adverse events (AEs). A multidisciplinary team (MDT) was responsible for determining the change of transfer to salvage liver resection. Hepatectomy was carried out under the hands of experienced surgeons.

### 2.3 Efficacy and safety assessment

The primary endpoint was overall survival (OS), and the secondary endpoints included adverse events (AEs), progression-free survival (PFS), disease control rate (DCR) and objective response rate (ORR) according to RECIST 1.1 criteria, respectively. Contrast-enhanced CT or MRI was completed in 2 cycles and evaluated by 2 radiologists with rich experienced in liver disease. If there was any discrepancy in the results, it was evaluated by another senior radiologist and resolved by consensus. Treatment efficacy was evaluated based on the response evaluation criteria in solid tumor (RECIST) version 1.1. ORR referred to complete response (CR) or partial response (PR) rate in patients. DCR referred to CR, PR or stable disease (SD) rate in patients. OS referred to the duration between admission and death of all causes. PFS referred to the duration between admission and death of all causes or disease progression. We recorded AEs throughout the treatment and rated them in line with CTCAE version 5.0 (Freites-Martinez et al., 2021).

### 2.4 Propensity score matching (PSM) analysis

This study used PSM analysis for reducing selection bias while balancing the baseline features between groups. Besides, variables that could affect the response to treatment and outcomes, such as age, gender, etiology, Child-Pugh class, Eastern Cooperative Oncology Group of Performance Status (ECOG-PS), AFP, number of intrahepatic lesions, tumor size, albumin-bilirubin (ALBI) grade, extrahepatic metastasis, and portal vein tumor thrombus (PVTT), were identified by stepwise logistic regression with forward selection methods performed by R software (version 4.0.3; R Foundation Inc., Vienna, Austria). To minimize bias, improve simplicity and interpretability, and reduce variance, we adopted the 1:1 nearest-neighbor matching algorithm at the 0.2 caliper.

### 2.5 Statistical analysis

Statistical analysis was performed with SPSS27.0 (SPSS, Chicago, IL, United States) and R software (version 4.0.3; R Foundation Inc., Vienna, Austria). The continuous-variable normality test was performed using the Shapiro-Wilk normality test. Continuous data were represented by median (interquartile range, IQR) or mean ± standard deviation, and analyzed by Mann-Whitney *U* test or student t test according to normality. Categorical data were represented by number and percentages and compared by fisher exact test or χ^2^ test. OS and PFS of time-to-event variables were estimated by Kaplan-Meier analysis, whereas log-rank test was conducted to analyze between-group differences. Risk factors related to survival were identified by univariable and multivariable Cox regression. Factors satisfying *p* < 0.1 from univariate regression were incorporated into multivariate regression. *p* < 0.05 (two-tailed) stood for statistical significance.

## 3 Results

### 3.1 Participants

**FIGURE 1:**
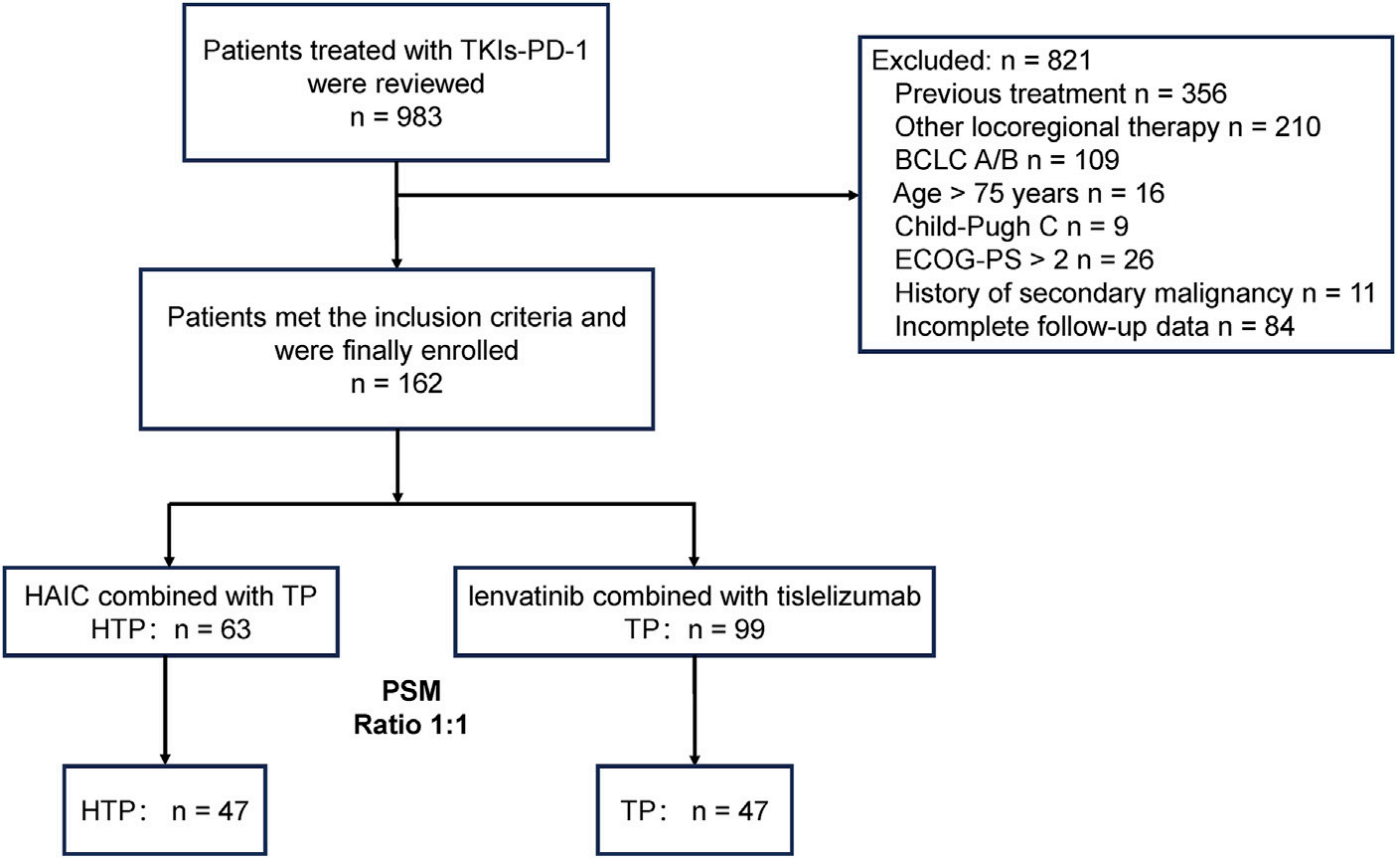
Patient selection flowchart. A patient might meet several exclusion criteria, but they were excluded only once from the uppermost criteria. PSM, propensity score matching; HTP, HAIC combined with lenvatinib and tislelizumab; TP, lenvatinib combined with tislelizumab; HAIC, hepatic arterial infusion chemotherapy; BCLC, Barcelona Clinic Liver Cancer; ECOG, Eastern Cooperative Oncology Group; PS, performance score.

Figure 1 displays the patient selection process. Altogether 162 advanced HCC patients with high tumor burden were enrolled, including 63 receiving the triple combination of HAIC plus lenvatinib and tislelizumab (HTP group), and the remaining 99 undergoing lenvatinib and tislelizumab without HAIC (TP group). Patients were followed up for a median period of 29.7 (95% Confidence interval [CI]: 18.4–38.9) and 24.9 (95% CI: 12.8–30.5) months till 30 June 2024, respectively. Age, ALBI class and extrahepatic metastasis were significantly different in both groups. PSM 1:1 ratio assigned 47 cases to every group for reducing the bias. Supplementary Table S1 shows the institutional distribution. Basic patient features were comparable in both groups following PSM (*p* > 0.2) (Table 1).

**TABLE 1:** Basic patient features before and following PSM.

Characteristic	Before PSM			Following PSM		
	TP n = 63	HTP n = 99	*p*	TP n = 47	HTP n = 47	*p*
Age (mean ± SD, year)	50 ± 12	45 ± 12	0.016	48 ± 11	47 ± 12	0.634
Age (year)			0.065			0.680
<50	45 (45.5%)	38 (60.3%)		23 (48.9%)	25 (53.2%)	
≥50	54 (54.5%)	25 (39.7%)		24 (51.1%)	22 (46.8%)	
Sex			0.174			0.748
Male	89 (89.9%)	52 (82.5%)		42 (89.4%)	41 (87.2%)	
Female	10 (10.1%)	11 (17.5%)		5 (10.6%)	6 (12.8%)	
ECOG-PS			0.070			>0.999
0	88 (88.9%)	61 (96.8%)		45 (95.7%)	45 (95.7%)	
1	11 (11.1%)	2 (3.2%)		2 (4.3%)	2 (4.3%)	
Etiology			0.799			>0.999
HBV	90 (90.9%)	58 (92.1%)		44 (93.6%)	44 (93.6%)	
Others	9 (9.1%)	5 (7.9%)		3 (6.4%)	3 (6.4%)	
Child-Pugh			0.115			>0.999
A	87 (87.9%)	60 (95.2%)		43 (91.5%)	44 (93.6%)	
B	12 (12.1%)	3 (4.8%)		4 (8.5%)	3 (6.4%)	
ALBI			0.044			0.680
1	39 (39.4%)	35 (55.6%)		24 (51.1%)	22 (46.8%)	
2	60 (60.6%)	28 (44.4%)		23 (48.9%)	25 (53.2%)	
AFP (μg/L)			0.652			0.836
<400	42 (42.4%)	29 (46.0%)		23 (48.9%)	22 (46.8%)	
≥400	57 (57.6%)	34 (54.0%)		24 (51.1%)	25 (53.2%)	
Intrahepatic lesion			0.054			0.472
Single	21 (21.2%)	22 (34.9%)		10 (21.3%)	13 (27.7%)	
Multiple	78 (78.8%)	41 (65.1%)		37 (78.7%)	34 (72.3%)	
Size (mean ± SD, cm)	12.2 ± 3.3	12.6 ± 3.4	0.367	12.4 ± 3.6	12.4 ± 3.3	0.931
Size (cm)			0.444			0.815
<10	29 (29.3%)	15 (23.8%)		13 (27.7%)	12 (25.5%)	
≥10	70 (70.7%)	48 (76.2%)		34 (72.3%)	35 (74.5%)	
PVTT			0.333			0.789
Presence	83 (83.8%)	49 (77.8%)		39 (83.0%)	38 (80.9%)	
Absence	16 (16.2%)	14 (22.2%)		8 (17.0%)	9 (19.1%)	
Extrahepatic metastasis			0.041			0.391
Presence	54 (54.5%)	24 (38.1%)		15 (31.9%)	19 (40.4%)	
Absence	45 (45.5%)	39 (61.9%)		32 (68.1%)	28 (59.6%)	

Continuous and categorical data are indicated by mean ± SD and n (%) separately.

P values were obtained by the two-sided Welch *t*-test and Pearson’s χ^2^ test.

PSM, propensity score matching; HTP, HAIC plus lenvatinib and tislelizumab; TP, lenvatinib plus tislelizumab; HAIC, hepatic arterial infusion chemotherapy; ECOG, eastern cooperative oncology group; PS, performance score; ALBI, albumin-bilirubin; AFP, alpha-fetoprotein; PVTT, portal vein tumor thrombus.

Prior to PSM, HTP group received median 4 cycles of HAIC (range: 2–8), 16 cycles of tislelizumab (range: 8–28), and 7.9 months of lenvatinib (range: 3.0–18.7), and patients in the TP group received median 12 cycles of tislelizumab (range: 6–23) and 5.1 months of lenvatinib (range: 3.0–15.2).

### 3.2 Tumor response

The treatment response was assessed before and following PSM according to RECIST 1.1 criteria (Table 2). Before PSM, HTP group showed increased ORR (58.7% versus 24.2%, *p* < 0.001) and DCR (88.9% versus 64.6%, *p* < 0.001), compared to TP group. After matching, HTP group also achieved higher rates of ORR (53.2% versus 17.0%, *p* < 0.001) and DCR (87.2% versus 61.7%, *p* = 0.004). Besides, the conversion rate to hepatectomy of HTP group was significant higher (before PSM: 1.0% versus 15.9%, *p* < 0.001; following PSM: 2.1% versus 17.0%, *p* = 0.015), as well.

**TABLE 2:** Therapeutic effect assessed through RECIST 1.1 criteria before and following PSM.

Response	Before PSM			Following PSM		
	TP (n = 99)	HTP (n = 63)	*p*	TP (n = 47)	HTP (n = 47)	*p*
Complete response	0 (0)	0 (0)		0 (0)	0 (0)	
Partial response	24 (24.2%)	37 (58.7%)		8 (17.0%)	25 (53.2%)	
Stable disease	40 (40.4%)	19 (30.2%)		21 (44.7%)	16 (34.0%)	
Progression disease	35 (35.4%)	7 (11.1%)		18 (38.3%)	6 (12.8%)	
Objective response rate	24 (24.2%)	37 (58.7%)	<0.001	8 (17.0%)	25 (53.2%)	<0.001
Disease control rate	64 (64.6%)	56 (88.9%)	<0.001	29 (61.7%)	41 (87.2%)	0.004
Conversion to hepatectomy	1 (1.0%)	10 (15.9%)	<0.001	1 (2.1%)	8 (17.0%)	0.015

Summary of optimal response.

Data are indicated by n (%).

*p* values were obtained by the two-sided χ^2^ test.

PSM, propensity score matching; RECIST, response evaluation criteria in solid tumors; HTP, HAIC plus lenvatinib and tislelizumab; TP, lenvatinib plus tislelizumab; HAIC, hepatic arterial infusion chemotherapy.

### 3.3 Survival endpoints

**FIGURE 2:**
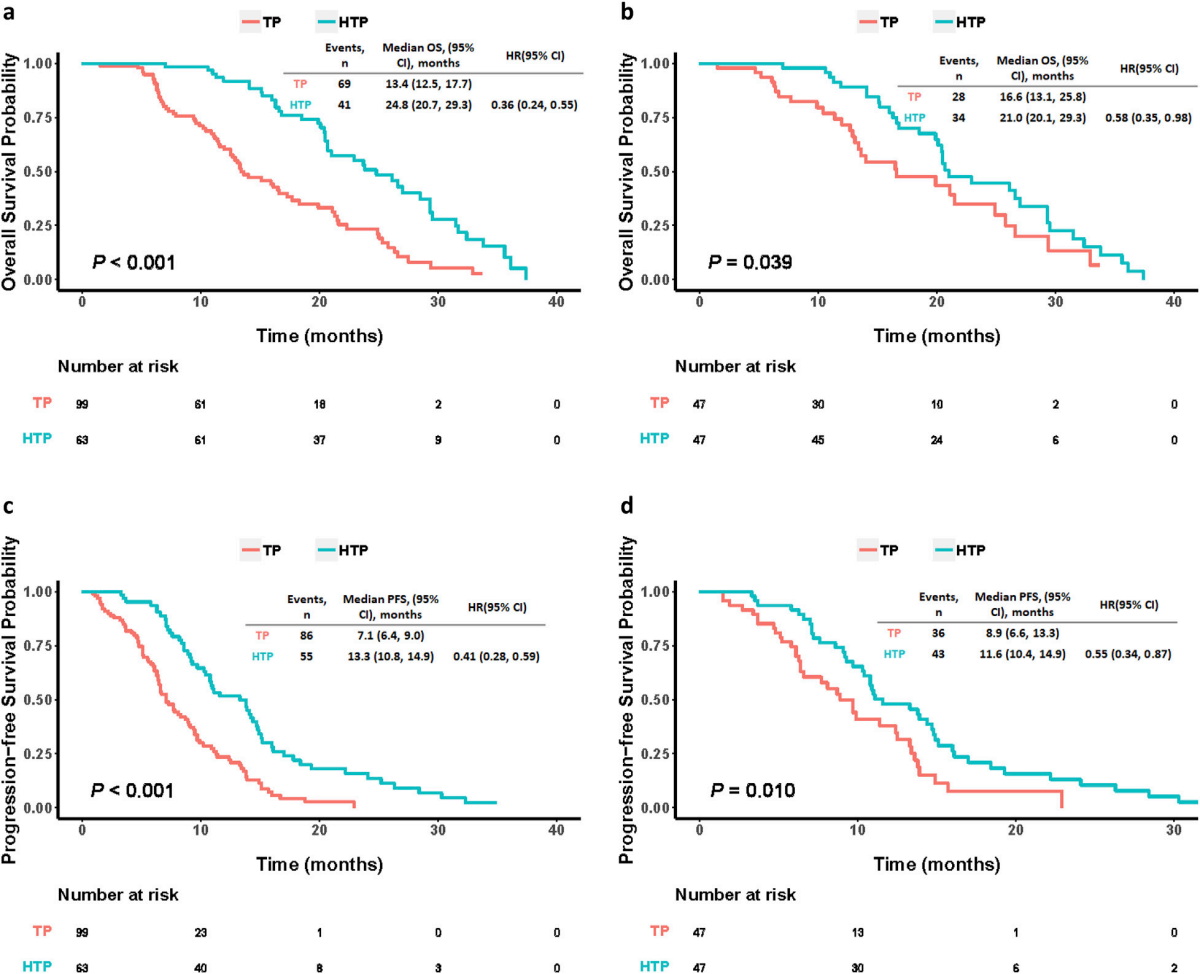
Kaplan-Meier curves comparing OS and PFS among patients who underwent HAIC plus lenvatinib and tislelizumab (HTP) or lenvatinib plus tislelizumab (TP) before **(A, B)** and after **(C, D)** PSM. *p* values were calculated using Log-rank test. PSM, propensity score matching; HTP, HAIC combined with lenvatinib and tislelizumab; TP, lenvatinib combined with tislelizumab; HAIC, hepatic arterial infusion chemotherapy; OS, overall survival; PFS, progression-free survival; HR, hazard ratio; CI, confidence interval.

Before PSM, there were 69 deaths (69.7%) from TP group and 41 (65.1%) from HTP group at the end follow-up time. HTP group had significantly prolonged OS (24.8 months, 95% CI: 20.7–29.3) compared with TP group (13.4 months, 95% CI: 12.5–17.7; HR: 0.36, 95% CI: 0.24–0.55, *p* < 0.001). Besides, HTP group also showed the markedly prolonged median PFS compared to TP group (13.3 months, 95% CI: 10.8–14.9 versus 7.1 months, 95% CI: 6.4–9.0; HR: 0.41, 95% CI: 0.28–0.59, *p* < 0.001). Of these 47 PSM pairs, HTP group also demonstrated evidently prolonged median OS than TP group (21.0 months, 95% CI: 20.1–29.3 versus 16.6 months, 95% CI: 13.1–25.8; HR: 0.58, 95% CI: 0.35–0.98, *p* = 0.039). Further, HTP group had prolonged median PFS compared with TP group (11.6 months, 95% CI: 10.4–14.9 versus 8.9 months, 95% CI: 6.6–13.3; HR: 0.55, 95% CI: 0.34–0.87, *p* = 0.010), as well (Figure 2).

### 3.4 Univariate and multivariate regression

This study carried out univariate and multivariate regression for identifying predictive factors for OS and PFS (Table 3). As revealed by multivariate Cox regression, triple combination treatment independently predicted the risk of OS and PFS. Besides, PVTT independently predicted the risk of OS. Etiology, extrahepatic metastasis and Child-Pugh class independently predicted the risk of PFS.

**TABLE 3:** Univariate and multivariate regression of predictive factors for survival post-treatment.

Variables	OS				PFS			
	Univariate regression		Multivariate regression		Univariate regression		Multivariate regression	
	HR (95% CI)	*P*	HR (95% CI)	*P*	HR (95% CI)	*P*	HR (95% CI)	*P*
Treatment (HTP)	0.36 (0.24–0.55)	<0.001	0.40 (0.26–0.61)	<0.001	0.41 (0.28–0.59)	<0.001	0.45 (0.31–0.65)	<0.001
Age (≥50 years)	0.95 (0.65–1.40)	0.811			0.89 (0.64–1.24)	0.494		
Sex (Male)	1.34 (0.73–2.44)	0.343			1.66 (1.01–2.74)	0.048	1.82 (0.85–3.16)	0.133
ECOG-PS (1)	2.17 (1.13–4.19)	0.021	1.62 (0.78–3.36)	0.199	1.38 (0.76–2.50)	0.289		
Etiology (Others)	1.50 (0.84–2.68)	0.174			1.70 (0.97–2.97)	0.064	2.32 (1.28–4.22)	0.006
Child-Pugh (B)	1.67 (0.89–3.13)	0.112			1.91 (1.07–3.41)	0.028	1.93 (1.07–3.49)	0.028
ALBI (2)	1.15 (0.79–1.68)	0.477			1.16 (0.83–1.62)	0.385		
Number (Multiple)	1.31 (0.85–2.02)	0.221			1.51 (1.03–2.22)	0.035	1.27 (0.86–1.90)	0.233
Size (≥10 cm)	1.18 (0.77–1.80)	0.451			0.72 (0.50–1.04)	0.083	0.75 (0.52–1.09)	0.132
AFP (≥400ug/L)	1.39 (0.94–2.06)	0.097	1.28 (0.84–1.96)	0.255	1.09 (0.78–1.52)	0.628		
PVTT (Presence)	2.14 (1.22–3.77)	0.008	1.89 (1.06–3.36)	0.030	1.00 (0.65–1.54)	0.985		
Metastasis (Presence)	0.98 (0.67–1.43)	0.903			1.52 (1.09–2.14)	0.015	1.44 (1.00–2.07)	0.047

Univariable and multivariable Cox regression conducted for identifying survival-related factors. Factors satisfying *p* < 0.1 upon univariate regression were incorporated for multivariate regression. *p* < 0.05 (two-sided) stands for statistical significance.

HTP, HAIC plus lenvatinib and tislelizumab; TP, lenvatinib plus tislelizumab; HAIC, hepatic arterial infusion chemotherapy; ECOG, eastern cooperative oncology group; PS, performance score; ALBI, albumin-bilirubin; AFP, alpha-fetoprotein; PVTT, portal vein tumor thrombus; OS, overall survival; PFS, progression-free survival.

### 3.5 Subgroup analysis

**FIGURE 3:**
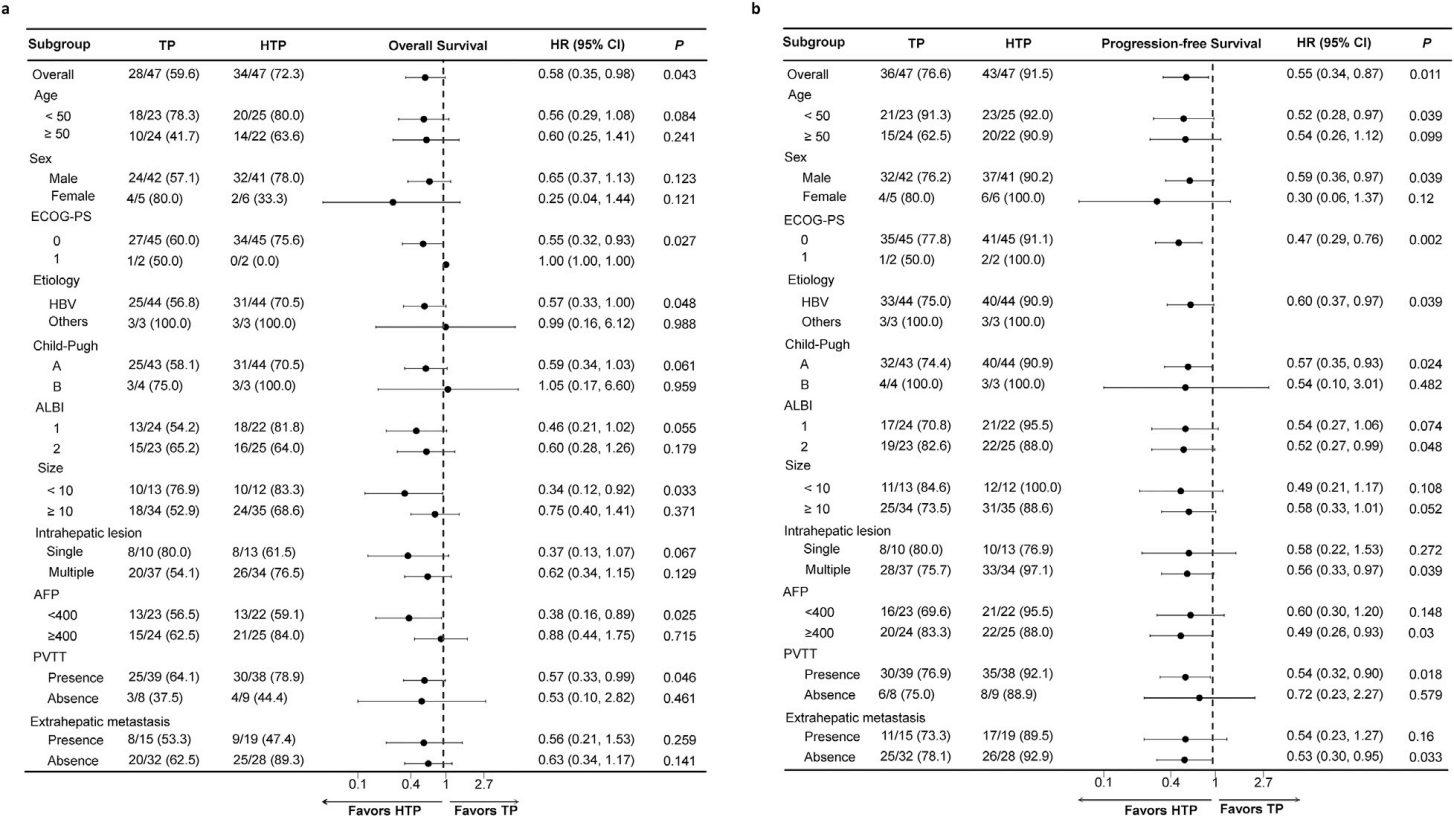
Forest plots based on OS **(A)** and PFS **(B)** of each subgroup. *p* values were calculated using Log-rank test. PSM, propensity score matching; HTP, HAIC combined with lenvatinib and tislelizumab; TP, lenvatinib combined with tislelizumab; HAIC, hepatic arterial infusion chemotherapy; OS, overall survival; PFS, progression-free survival; HR, hazard ratio; CI, confidence interval; ECOG, Eastern Cooperative Oncology Group; PS, performance score; ALBI, albumin-bilirubin; AFP, alpha-fetoprotein; PVTT, portal vein tumor thrombus.

This work drew forest plots for illustrating comparison among subgroups (Figure 3). As for the OS (Figure 3A) and PFS (Figure 3B), HTP group exhibited more beneficial effects in nearly every subgroup in comparison with TP group, indicating the effectiveness of HAIC, lenvatinib and tislelizumab combination therapy on advanced HCC patients with high tumor burden in each subgroup.

### 3.6 Progression reason analysis

Altogether 4 ways for tumor progression were detected, including extrahepatic metastasis, intrahepatic metastasis, local lesion progression, and death. For HTP and TP groups, there were respective 55 and 86 patients who have progressed at the cut-off follow-up time, and the proportion of four ways were 32.6%, 36.0%, 22.1% and 9.3% versus 21.8%, 27.3%, 21.8% and 29.1%. HTP group had lower proportion of intrahepatic metastasis and lesion progression compared with TP group (Supplementary Figure S1).

### 3.7 Subsequent treatments

At the end of follow-up, 78 and 39 patients in the HTP and TP group, respectively, experienced tumor progression (except for those due to death). Additionally, 55 (70.5%) and 32 (82.1%) patients in the two groups, separately, underwent subsequent treatment. In TP and HTP groups, HAIC combined with lenvatinib plus tislelizumab (34.5%), and TACE combined with lenvatinib and tislelizumab (31.3%) were the most frequently adopted subsequent treatments. TACE and HAIC remained the preferred options for locoregional treatment, while atezolizumab plus bevacizumab and regorafenib were the preferred options for systemic treatment after progression (Supplementary Table S2).

### 3.8 Safety

As exhibited in Table 4, overall incidence rates of AEs in HTP and the TP group were 82.5% and 80.1%, respectively. In HTP group, AEs including decreased appetite (77.8%), neurologic toxicity (60.3%), and leukopenia (57.1%) occurred at the highest frequency, whereas severe AEs (grade 3/4) with the highest occurrence frequency included abdominal pain (7.9%), diarrhea (7.9%), and proteinuria (7.9%). In TP group, AEs like hypertension (52.5%), decreased appetite (41.4%), and elevated AST (33.3%) occurred at the highest frequency, and severe AEs with the highest frequency included proteinuria (10.1%), diarrhea (7.1%) and hypertension (7.1%). Though the overall incidence of any grade and severe AEs was higher in HTP group, there were no significant difference between the two groups. Obviously, HAIC-related (e.g., hypertension and hand-foot skin reaction) AEs was significantly higher in HTP group, but these AEs could be managed, and no treatment-related death happened.

**TABLE 4:** Treatment-associated adverse events.

Adverse events	Any grade			Grade 3/4		
	HTP (n = 63)	TP (n = 99)	*P*	HTP (n = 63)	TP (n = 99)	*P*
Overall incidence	52 (82.5%)	80 (80.1%)	0.782	22 (34.9%)	37 (37.4%)	0.752
Abdominal pain	35 (55.6%)	18 (28.6%)	0.000	5 (7.9%)	1 (1.0%)	0.033
Nausea	29 (46.0%)	21 (21.2%)	0.001	3 (4.8%)	2 (2.0%)	0.378
Diarrhea	31 (49.2%)	24 (24.2%)	0.001	5 (7.9%)	7 (7.1%)	0.837
Fever	26 (41.3%)	4 (4.0%)	0.000	1 (1.6%)	2 (2.0%)	1.000
Decreased appetite	49 (77.8%)	41 (41.4%)	0.000	2 (3.2%)	3 (3.0%)	1.000
Rush	13 (20.6%)	29 (29.3%)	0.220	2 (3.2%)	4 (4.0%)	1.000
Fatigue	25 (39.7%)	19 (19.2%)	0.004	2 (3.2%)	3 (3.0%)	1.000
Hypoproteinemia	16 (25.4%)	21 (21.2%)	0.536	2 (3.2%)	2 (2.0%)	0.643
Elevated bilirubin	19 (30.2%)	25 (25.3%)	0.494	3 (4.8%)	4 (4.0%)	1.000
Elevated ALT	31 (49.2%)	25 (25.3%)	0.002	2 (3.2%)	1 (1.0%)	0.561
Elevated AST	29 (46.0%)	33 (33.3%)	0.105	1 (1.6%)	1 (1.0%)	1.000
Hypothyroidism	4 (6.3%)	6 (6.1%)	1.000	1 (1.6%)	1 (1.0%)	1.000
Neurologic toxicity	38 (60.3%)	6 (6.1%)	0.000	4 (6.3%)	0 (0)	0.022
Leukopenia	36 (57.1%)	17 (17.2%)	0.000	3 (4.8%)	1 (1.0%)	0.300
Thrombocytopenia	25 (39.7%)	29 (29.3%)	0.171	4 (6.3%)	6 (6.1%)	1.000
Hypertension	20 (31.7%)	52 (52.5%)	0.009	3 (4.8%)	7 (7.1%)	0.742
Hand-foot skin reaction	18 (28.6%)	29 (29.3%)	0.921	3 (4.8%)	7 (7.1%)	0.742
Dysphonia	5 (7.9%)	14 (14.1%)	0.231	0 (0.0%)	1 (1.0%)	1.000
Proteinuria	11 (17.5%)	28 (28.3%)	0.116	5 (7.9%)	10 (10.1%)	0.643
Bleeding (gingiva)	4 (6.3%)	8 (8.1%)	0.767	1 (1.6%)	3 (3.0%)	1.000
Joint pain	11 (17.4%)	25 (25.3%)	0.245	3 (4.8%)	6 (6.1%)	1.000
Immunity-related AEs	9 (14.3%)	17 (17.2%)	0.626	2 (3.2%)	5 (5.1%)	0.707

Data are represented by n (%).

*p* values were determined by two-sided χ^2^ test.

HTP, HAIC combined with lenvatinib and tislelizumab; TP, lenvatinib combined with tislelizumab; HAIC, hepatic arterial infusion chemotherapy; ALT, alanine transaminase; AST, aspartate aminotransferase.

## 4 Discussion

The present multicenter clinical study is the first to examine whether HAIC plus lenvatinib and tislelizumab was as effective and safe as lenvatinib plus tislelizumab for advanced HCC with high tumor burden, and the triple combination therapy significantly improved survival and tumor response compared with the dual combination therapy. Besides, PSM was conducted for eliminating group heterogeneities, a relatively large sample size was included among diverse centers, and long-time follow-ups were performed. At last, no matter in PSM cohort or the whole cohort, HTP group showed significantly prolonged OS and PFS comparing to TP group. Noteworthily, triple combination therapy induced the increased AEs rate, while efficient measures were adopted for mitigating AEs.

According to current guidelines, locoregional therapy, mainly referring to TACE, has been recommended to be the first-line therapy for immediate-stage HCC, while systemic treatment remains the preferred option in advanced stage HCC (Singal et al., 2023; Vogel and Martinelli, 2021). Although the dual immunotherapies atezolizumab-bevacizumab and durvalumab-tremelimumab have been shown to result in better OS and PFS than sorafenib, lenvatinib remained the first-line choice for PVTT patients in China, giving the rather high cost of the dual immunotherapies and the prevalent risk of gastric bleeding due to cirrhosis. However, in the phase 3 randomized controlled study of LEAP 002, lenvatinib plus pembrolizumab failed to meet the pre-specified statistical significance on OS, even though the combination therapy achieved the longest median OS time ever reported in first-line HCC studies. Hence, it is worth noting that locoregional treatment and systemic treatment should complement each other rather than be mutually exclusive, especially for those with high-risk factors, including high tumor burden or extrahepatic metastasis. Locoregional therapy can reduce the tumor burden in a relatively short time, while systemic therapy can effectively control the intrahepatic and extrahepatic lesions in a relatively longer period, resulting in an enhanced synergistic anti-tumor effect (Cappuyns et al., 2024; Brown et al., 2023). As reported, an increasing number of clinical trials have demonstrated that locoregional therapy, including TACE or HAIC, combined with TKIs and/or PD-1 inhibitors possessed favorable outcomes in advanced HCC patients, comparing to monotherapy or dual combination therapy (Kulik and El-Serag, 2019). As reported in a phase II clinical study (TRIPLET), HAIC combined with camrelizumab and apatinib for HCC in BCLC C stage demonstrated encouraging results and manageable safety. Over 50% of the enrolled patients had high risk factors, including Vp3/4 or extrahepatic metastasis. The median PFS was 10.38c months, and the ORR and DCR were 77.1% and 97.1%, respectively (Chen et al., 2023), consistent with our study.

Some rationales were proposed for combining HAIC rather than TACE as the locoregional treatment in the study. First, previous studies have demonstrated that comparing with TACE, FOLFOX-HAIC results in favorable tumor response and superior outcomes for advanced HCC due to the direct delivery high-dose chemotherapeutic drugs into tumor via hepatic artery (Li et al., 2021). As a result, Chinese guidelines have recommended HAIC rather TACE as one of first-line options for advanced HCC, particularly for patients with high tumor burden (Xie et al., 2023). Second, TACE can induce hypoxia in the tumor microenvironment, which may activate hypoxia-inducible factors (HIFs) and promote tumor survival, angiogenesis, and subsequent recurrence (Gai et al., 2020). Third, many embolization particles are required for embolizing large-sized HCC, probably leading to an increased risk of hepatic functional reserve deterioration, nontarget embolization, and postembolization syndrome (Roehlen et al., 2023). Finally, total embolization is not easy for large-sized HCC due to extrahepatic collateral arteries (Li et al., 2022).

We also carried out subgroup analyses on OS and PFS according to different factors. HTP group showed the clinical benefits for almost all the subgroups. Nonetheless, broad 95% CI ranges were observed in females, patients of Child-Pugh class B, and those without HBV infection, probably associated with the small sample size in each subgroup.

When analyzing various endpoints to PFS, HTP group had obviously lower proportion of intrahepatic metastasis and local lesion progression comparing to TP group. These findings suggested that HAIC, as a locoregional approach, could play an important role in controlling intrahepatic and local recurrence, and the triple combination therapy could exert the synergistic anticancer efficacy. Expectedly, intrahepatic and local recurrence mainly restricted the survival benefits of systemic therapy. Therefore, the combination with HAIC probably further improved prognosis (Yuan et al., 2023).

The ration of subsequent therapy was higher in HTP group (82.1% versus 70.5%), suggesting that treatment following progression might be conducted at a higher frequency among patients receiving HAIC plus lenvatinib and tislelizumab. This may be explained from two aspects. First, triple combination therapy provided superior efficacy, improving the compliance of patients. Second, triple combination therapy significantly prolonged survival, providing more opportunities for subsequent treatments.

In addition to favorable outcomes, HAIC combined with lenvatinib and tislelizumab increased AEs rate to some extent. The higher HAIC-related AEs incidence took place, consistent with those in previous trials of HAIC. However, those AEs could be controllable using corresponding supporting medications, without aggravating the condition or treatment discontinuation. HTP group had increased AEs incidence relative to locoregional therapy or systemic therapy in the previous studies (Kudo et al., 2018; Qin et al., 2023; Llovet et al., 2023; Lyu et al., 2018a), the reasons of which may be that the enrolled patients in the study were with worse baseline levels, and the systemic therapy aggravated the toxicity of HAIC. Abdominal pain resulting from arteria vasospasm in oxaliplatin infusion was also a common HAIC-specific AE. So far, abdominal pain cannot be effectively avoided, with the exception of the prescription of pain/spasm relieving agents or slowing down oxaliplatin infusion (Wu et al., 2021; Proietti et al., 2007). Collectively, HAIC combined with lenvatinib and tislelizumab showed acceptable safety and tolerability.

Certain limitations should be noted in the present work. First, due to the retrospective nature, selection bias could not be avoided. Though PSM was conducted for minimizing between-group heterogeneities, there were still endogenous differences due to unmeasured confounders, population differences, temporal bias, exclusion of non-matched cases, and information bias. Consequently, more prospective randomized controlled studies should be conducted for validating our results. Second, since the study primarily included HBV-positive individuals, it is important to note that HCC in patients with HBV infection can differ biologically and clinically from HCC in individuals with other etiologies, such as hepatitis C virus (HCV) or non-alcoholic fatty liver disease (NAFLD). The treatment responses, prognosis, and underlying mechanisms of HCC may vary across these different patient populations. It should acknowledge that the findings may not be fully applicable to non-HBV patients, especially in regions where HCV or NAFLD-related HCC is more common. Third, the patient cohort being sourced mainly from Chinese medical centers is another important limitation. The genetic, environmental, and healthcare-related factors in China may differ from those in other countries or regions, such as Europe or North America. These differences could impact treatment response and outcomes. It is important to state that the study’s results may not be directly applicable to populations in other parts of the world, particularly where the prevalence of HBV and other risk factors for HCC may differ significantly. The study should call for further validation in more diverse cohorts from different geographic regions to confirm the external validity of the findings. Finally, we just enrolled cases undergoing tislelizumab treatment, and further exploration is warranted for determining if the same results are attained using other PD-1 inhibitors.

## 5 Conclusion

In conclusion, comparing to lenvatinib plus tislelizumab, HAIC plus lenvatinib and tislelizumab achieves markedly improved OS, PFS, and ORR for advanced HCC with high tumor burden, and this combination treatment is safe. Before prospective results are revealed, our results support applying the triple combination treatment for advanced HCC with high tumor burden.

## Data Availability

The original contributions presented in the study are included in the article/Supplementary Material, further inquiries can be directed to the corresponding authors.
