# Supplementary material for: Efficiency and safety of HAIC combined with lenvatinib and tislelizumab for advanced hepatocellular carcinoma with high tumor burden: a multicenter propensity score matching analysis
Source: Front Pharmacol. 2025 Jan 7;15:1499269. doi: 10.3389/fphar.2024.1499269 (PMC11747745; doi:10.3389/fphar.2024.1499269)
Supplement: Supplementary file 1 [file DataSheet1.docx]

Supplementary Material

# Supplementary Tables and Figure

**Table S1** The patients enrolled in each center

| **Center** | **Before matching** | |  | | **After matching** | | |
| --- | --- | --- | --- | --- | --- | --- | --- |
|  | **HTP**  **(n = 99)** | **TP**  **(n = 63)** | |  | | **HTP**  **(n = 47)** | **TP**  **(n = 47)** |
| Binzhou Medical University Hospital | 39 (39.4%) | 27 (42.9%) | |  | | 20 (42.6%) | 21 (44.7%) |
| Sun Yat-sen Memorial Hospital | 35 (35.4%) | 22 (34.9%) | |  | | 15 (31.9%) | 17 (36.2%) |
| Sun Yat-Sen University Cancer Center | 25 (25.2%) | 14 (22.2%) | |  | | 12 (25.5%) | 9 (19.1%) |

Values are presented as n (%).

HTP, HAIC plus lenvatinib and tislelizumab; TP, lenvatinib plus tislelizumab; HAIC, hepatic arterial infusion chemotherapy.

**Table S2** Subsequent treatment in each group

|  | TP (n=78) | HTP (n=39) |
| --- | --- | --- |
| Received subsequent treatment | 55 (70.5%) | 32 (82.1%) |
| HAIC + lenvatinib + tislelizumab | 19 (34.5%) | 5 (15.6%) |
| TACE + lenvatinib + tislelizumab | 8 (14.5%) | 10 (31.3%) |
| HAIC + atezolizumab + bevacizumab | 5 (9.1%) | 3 (9.4%) |
| Atezolizumab + bevacizumab | 3 (5.5%) | 3 (9.4%) |
| Regorafenib | 4 (7.4%) | 1 (3.1%) |
| Regorafenib + tislelizumab | 7 (12.7%) | 6 (18.7%) |
| HAIC + regorafenib | 2 (3.6%) | 0 (0) |
| TACE + regorafenib | 2 (3.6%) | 1 (3.1%) |
| HAIC + regorafenib + tislelizumab | 3 (5.5%) | 1 (3.1%) |
| TACE + regorafenib + tislelizumab | 2 (3.6%) | 2 (6.3%) |
| Best support care | 20 (25.6%) | 6 (15.4%) |

Values are presented as n (%).

HTP, HAIC plus lenvatinib and tislelizumab; TP, lenvatinib plus tislelizumab; TACE, transarterial chemoembolization; HAIC, hepatic arterial infusion chemotherapy.

**
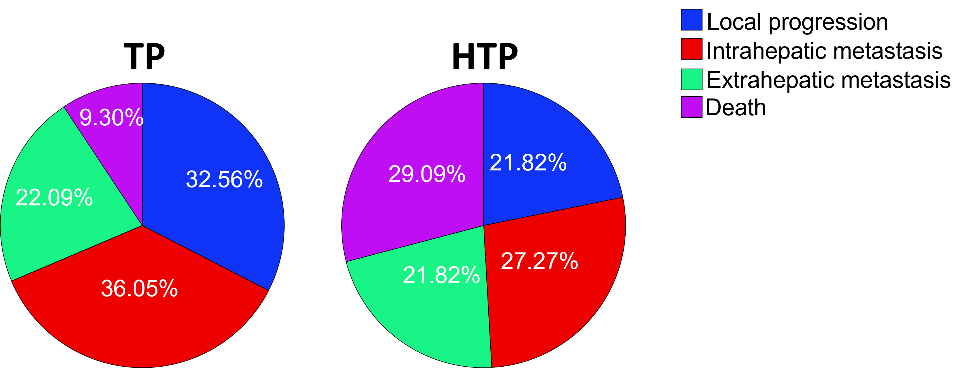
**

**Figure S1.** Progression patterns in both groups.

HTP, HAIC plus lenvatinib and tislelizumab; TP, lenvatinib plus tislelizumab; HAIC, hepatic arterial infusion chemotherapy.
